# Transvestism Recognized in Ehlers-Danlos Syndrome: Report of Two Cases

**DOI:** 10.1155/2019/7472301

**Published:** 2019-08-06

**Authors:** Hiroki Ishiguro, Naomi Honobe, Takefumi Suzuki, Mariko Tamai, Takaya Nakane

**Affiliations:** ^1^Department of Neuropsychiatry and Clinical Ethics, Graduate School of Medicine, University of Yamanashi, Japan; ^2^Genetic Disease Medical Center, University of Yamanashi Hospital, Japan; ^3^Department of Fundamental Nursing, University of Shinshu, School of Health Sciences, Japan; ^4^Department of Pediatrics, Graduate School of Medicine, University of Yamanashi, Japan

## Abstract

Ehlers-Danlos syndrome (EDS) comprises a series of rare hereditary connective tissue diseases characterized by musculoskeletal, skin, and cardiovascular involvements. EDS may be associated with physical as well as psychological pain that can lead to psychiatric problems. EDS imposes substantial psychological burden on patients, and recent large-scale studies have suggested that patients with EDS have a higher risk of mood disorders than the general population. To the best of our knowledge, we describe, for the first time, the cases of two Japanese patients with EDS complicated with mood disorders who secondarily developed transvestism that was judged strongly related to early stressful situations through childhood and adolescence. The first case was of a man in his mid-30s and the second of a woman in her late 20s. We report on detailed psychosocial data to further discuss the medical management and genetic counseling of such infrequent but challenging conditions. Physicians are advised to be aware of various potential psychological and psychiatric issues that may accompany EDS.

## 1. Introduction

Doctors with dual certification as clinical geneticists and psychiatrists have the special opportunity to treat patients with Ehlers-Danlos syndrome (EDS) complicated with mental disorders in Japan. EDS comprises a series of hereditary connective tissue diseases and typically involves musculoskeletal, dermatological, and cardiovascular problems. While EDS had been classified into more than 10 types in the past, it was reclassified in 2017 according to symptomatic features: classical (types I and II), hypermobility (type III), vascular (type IV), kyphoscoliosis (type VIA), arthrochalasia (type VIIA and VIIB), dermatosparaxis (type VIIC) types, and rare disease types observed in low numbers of families [1]. EDS has an overall prevalence of 1/5000, and multiple causative genes involved in each type have been identified, although some implicated genes remain elusive [2]. The inherited form is mostly autosomal dominant in the classical, hypermobility, and arthrochalasia types, while it is autosomal recessive in the dermatosparaxis and kyphoscoliosis types. EDS mainly involves physical symptoms and there is little evidence of central nervous system involvement. The classification for EDS does not include specific disease types associated with intellectual developmental disorder or with psychiatric symptoms.

Patients with EDS reportedly are at a relatively high risk of mental disorders, such as anxiety disorder and mood disorders accompanied by pain including headache, muscle pain, neuralgia, abdominal pain, and malaise [3–5]. Depressive symptoms in patients with EDS were first reported in 2003[6]. Because patients with depression frequently experience pain [7, 8], some of the pain reported by patients with EDS could be either attributable to physical symptoms, due to dislocation, for instance, or to depression. Additionally, chronic pain in such patients may lead to negative emotions due to psychological burden [3]. Furthermore, a large-scale study on the Swedish population found that patients with EDS have a higher risk of both mood disorders and developmental disorders than the general population [4].

Here, we report on two rare cases of EDS complicated with transvestism as a psychological symptom. Treatment of mood disorders was rendered challenging due to major contribution of pain caused by EDS. According to the Diagnostic and Statistical Manual of Mental Disorders, 5th Edition (DSM-5) criteria, gender dysphoria in adolescents and adults is characterized by (A) a marked incongruence between the experienced gender and assigned gender and (B) the condition associated with significant distress or impairment in social, occupational, or other important areas of functioning. For children with gender dysphoria, a strong preference for cross-dressing is listed as one of the criteria in the DSM-5, while it is not mandatory clause. The children do not prefer the clothing and hairstyles socially expected for the assigned gender and have intense negative reactions when their parents attempt to have them behave in manners typical of their assigned gender at birth [9]. Although a twin study by Coolidge et al. suggested that gender dysphoria may be heritable by 62% [10], careful investigation for possible emotional distress is also needed.

The term transvestism, from the Latin “trans” (across, over) and “vestitus” (dress and dressed, clothed), was introduced by Magnus Hirschfeld in his book [11]. Transvestism, also termed cross-dressing, is not itself a disorder per se; it develops during childhood or adolescence, and most individuals who cross-dress are heterosexual. These individuals do not normally appear anxious or concerned of the cross-dressing behaviors because they use this behavior to reduce anxiety, escape into a fantasy world, or try to cope with their vulnerabilities. Conversely, transvestic disorder is a psychiatric condition, causing psychological distress when they engage in such actions. Clinically, most of cross-dressers are not diagnosed with transvestic disorder. When cross-dressing behavior is not accompanied by feelings of anxiety or concern, it is not diagnosed as transvestic disorder. An individual must meet the following DSM-5 criteria in order to be diagnosed with transvestic disorder: (a) the individual experiences intense sexual arousal from cross-dressing, as manifested by fantasies, urges, or acts, for at least 6 months and (b) these fantasies, urges, and acts cause clinically significant distress or impairment in social, occupational, or other important areas of life.

Informed consent was obtained from both patients to compose the case report with assurances of preserving patient anonymity and privacy.

## 2. Case Presentation

The first case was of a Japanese man in his mid-30s. His major psychiatric issues included bipolar I disorder, intellectual developmental disorder, and transvestism. Although formal genetic testing was not performed, he showed typical clinical presentation of EDS: skin hyperextensibility, generalized joint hypermobility (which satisfied the two major criteria, as set in 2017), soft and doughy skin including the nose septum and auricular, flexible flatfoot, epicanthal folds on the tongue (which satisfied three minor criteria), and phlebitis in his legs (as an additional symptom). His medical history included fetal asphyxia, pediatric asthma, congenital hip arthritis, for which he had undergone two surgeries at 5 and 20 years of age, and hepatitis C caused by blood transfusion that was performed at the second surgery. Since there was no history of interferon therapy during the 10 years before the first visit to a psychiatrist (at year X), the possibility is remote that he had interferon-associated psychiatric disturbances. His premorbid personality traits included nervousness, obstinance, and incoordination. He had no relatives with EDS. The patient was the second son among three siblings. His father had bipolar disorder and committed suicide when the patient was 13 years old.

He was evaluated for intellectual developmental disorder in his adulthood twice at years X+1 and X+6. His verbal intelligence quotient (IQ) was found to be 67 and 77, performance IQ 69 and 49, and full IQ 66 and 62, respectively. In addition, magnetic resonance imaging scans revealed cavum septi pellucidi (CSP) and small infarct lesions in the cerebral white matter. The psychological examinations, including the Rorschach test and the Minnesota Multiphasic Personality Inventory suggested a low degree of autonomy or insight but no abnormality in recognizing himself as male gender.

The patient had experienced sexual trauma, as he had been molested by his father in his elementary-school years. He also experienced bullying by classmates because of his poor academic performance at that time. During this difficult time, the father committed suicide. After his father's death, the patient started exhibiting cross-dressing behavior. There was no particular evidence indicating a correlation between his transvestic behavior and his father's death.

Despite his poor academic performance, he was able to graduate from senior high school. After spending 1 year at a training school for persons with disabilities, he secured a clerk position at a general corporation as an employee with disabilities. He first visited a psychiatrist (at year X) because he had started experiencing paranoia in the workplace. The symptom subsided with a low dose of haloperidol but at year X+3, paranoia reappeared along with irritation, insomnia, loss of motivation, hypochondriac symptoms, and depressive mood as his job became more demanding. One month after the relapse, his mood changed to mania with insomnia, anger, irritation, and increased appetite and libido. The patient recovered with mood stabilizers, antipsychotics, and a 6-month sick leave from his job. However, he again relapsed 3 months after remission was achieved. Since then, his affect has remained cyclic while receiving medical treatment either as an outpatient or inpatient.

The intellectual developmental disorder in this case could have possibly been caused by several factors: (1) EDS, (2) past history of fetal asphyxia, (3) presence of CSP, and (4) others. CSP reportedly occasionally causes intellectual developmental disorder [12], emotional mood disorders, and schizotypal symptoms in adulthood [13]. Although remarkably enlarged CSP (anteroposterior diameter ≥ 6 mm) is associated with serious mental illnesses such as schizophrenia [14, 15], many patients with less prominent CSP remain asymptomatic [15]. The association between CSP and bipolar disorder is generally weaker than that with schizophrenia [16]; the CSP in this case was not enlarged to the extent that could account for all symptoms.

While his cross-dressing habit began in childhood, first in private in his room, and persisted in public through adulthood, his behaviors and symptoms did not meet the DSM-5 criteria of gender dysphoria. Although he used complete female attire, including upper and lower undergarments, he did not apply female make up on his face and did not look himself in the mirror when wearing female clothes; moreover, his desire to wear female clothes expired when he received psychological support from a nurse at the Company Health Office. His desire was primarily to wear female clothes and his consideration for a sex-change was secondary to feelings of slight guilt and shame that accompanied the desire for cross-dressing. He did not have intense sexual arousal, distress, or impairment in social life, and thus did not meet the DSM-5 criteria for transvestic disorder.

Regarding the relationship between transvestism and mood disorders, cross-dressing is reportedly more remarkable during the manic phase and becomes inconspicuous during the remission phase [17]. In this case, his desire to cross-dress remained consistent in the remission phase of the bipolar disorder, and the cycle of mood symptoms did not affect the degree of cross-dressing to a considerable degree. We considered that his symptoms could be attributable to (a) his mother's overprotectiveness and excessive interference due to his intellectual developmental disorder and EDS, (b) conflict with his older brother when he was in the manic phase, and (c) maladaptation in the job environment and failures in the establishment of human relationships. While patients with intellectual developmental disorder may exhibit the so-called pseudo-transvestism used as a means to fantasize women during masturbation [18], cross-dressing was the objective per se for this patient. Additionally, because he was more interested in looking at pictures of himself wearing female underwear than in changing his gender, his behavior could have been obsessive/compulsive to some degree in the sense that he was overwhelmingly driven to wear female clothes; by doing so, he considered that he could escape into a fantasy world where he could cope with his desire. Anupama et al. [19] reported three cases of patients who cross-dressed; two had intellectual developmental disorder and one had obsessive-compulsive disorder. Meanwhile, Bowler et al. [20] proposed that patients with intellectual developmental disorder tended to engage in cross-dressing, as it was difficult for them to communicate with the opposite sex, both personally and socially. Although cross-dressing is more frequently observed in patients with intellectual developmental disorder than in the general population, their symptoms subside when they are involved in a relationship, while their preference for brilliant clothing continues.

The family environments during childhood have been carefully studied in relation to transvestism; coldness, high pressure, or weak presence with only few emotional exchanges all have been associated with distorted father images in these patients [21–23].

The second case was of a Japanese woman in her late 20s. Her major diagnoses were dissociative identify disorder, gender dysphoria, dysthymia, and anorexia nervosa (restriction type). She had classical EDS with skin hyperextensibility and atrophic scarring, generalized joint hypermobility (which satisfied the second major criterion based on the 2017 criteria), easy bruising, soft and doughy skin, skin fragility and traumatic splitting, complications of joint hypermobility such as pain, and family history (which satisfied the fifth minor criterion). The final diagnosis was straightforward even without genetic testing. She did not have notable past medical history and had nervousness as her premorbid personality trait. Her monozygotic twin sister also had EDS, and except for her twin sister, she also had an older brother; none of her other relatives had EDS or diagnosed psychiatric disorders including gender dysphoria and dissociative identity disorder.

The patient was brought up as older sister in contrast with that another twin sister was treated as younger sister in their family, although they are monozygotic twins. She was prone to injuries and dislocations and had led an infirm life since childhood. In elementary school, she was frequently bullied for her facial scars and suffering from dysthymic status. In addition, as far as the patient could recollect, she started to feel uncomfortable wearing women's clothes in her early elementary-school years. Her mother reacted to the situation by overprotection, imposing rules to prohibit the patient from playing outdoors or behaving as a boy and restricting her behavior inside the house. Compared with her mother's parental control, her father was judgmental against her “laziness” because she was unable to overcome her dysthymic personality/character and secure regular employment after graduating from high school.

She was aware of having dissociative identity disorder since childhood based on her recollections of first experiencing a personality change at the age of 5 years. The original personality (A) corresponded to a patient with anorexia nervosa and was mainly dormant. She was aware of two male personalities present most of the time; personality (B) who oversaw meals and personal activities and personality (C) who oversaw contact with others (including during consultation with the psychiatrist). The replacement personalities appeared depending on the circumstances; several male personalities, including (B) and (C), dominated during housekeeping, eating, and social encounters, while the female personalities were likely to surface when the patient was required to dress and behave in a female manner during her school years and, currently, those personalities appear to be integrated as personality (D), emerging only when the patient is concerned with feminine hygiene during bathing and menstruation. In the past, she presented with a larger number of personalities, but they were consolidated to the relatively few personalities mentioned above, presumably as a coping mechanism in an effort to maintain wellness.

When limb movement paralysis and inability to open her eyes appeared at the age of mid-20s, a physical examination was performed at a hospital as her first major psychiatric consultation (at year Y). Meanwhile, no physical abnormalities were detected and she was diagnosed with dissociative movement disorder. Since then, she has been occasionally treated as an outpatient at the psychiatric department of the university hospital. Despite her main complaint of excessive dietary restriction and self-induced vomiting based on her desire to lose weight, psychiatric treatment was intermittent. She had strong desire for social support, self-acceptance, and integration of a male identity. To fulfil what she considered were the requirements of a male identity, she habitually used male public restrooms. Finally, she received a first psychiatric diagnosis of gender dysphoria based on the DSM-5 criteria, with the disorder involving years of marked incongruence between the self-referenced and assigned genders. At year Y+2, she relocated without her parents' permission to the house of a friend with dissociative identity disorder who she knew through the Social Network Service. She was then transferred to another hospital located near her home at year Y+3, where the psychiatric diagnosis of gender dysphoria was confirmed based on the DSM-5 criteria.

The difference in intellectual level between the two patients may have led to different phenotypes, hypochondriac behavior in Case 1 and dissociative identity disorder in Case 2. Patients with dissociative identity disorder often experience considerable stress during early and late childhood, which may include (a) bullying from schoolmates and siblings, (b) restrictions in self-expression by overly controlling parents, (c) child abuse including neglect, and (d) mental trauma caused by accidents or other incidents. Factors (a) to (c), as well as (d), render individuals susceptible to trauma and were present and constituted high risk of dissociative identity disorder in Case 2. The specific personalities of dissociative identity disorder were deemed to stem from the clinical features of EDS. Primary personality (A) had anorexia nervosa, possibly influenced by insufficiency of maternal separation from early childhood because of her mothers' overprotectiveness and excessive interference driven by her concern for the high risk of trauma associated with EDS. Eating disorder is a symbolic disease mainly observed in women, and the patient attempted to deny her femininity through her gender dysphoria. Although she reported experiencing a strong sense of discomfort and disgust for being treated as a woman, she also understood the risks of surgery because of her EDS and did not consider a sex-change operation. As a compensatory mechanism against these feelings, the male personalities (B) and (C) became dominant, overseeing meals to counter anorexia nervosa, which is a symbol of femininity, controlling physical health, and acting as a form of psychological coping mechanism to protect the patient against the discomfort of gender dysphoria. It is difficult to discern whether anorexia nervosa or gender dysphoria was the primary disorder. To summarize, the prolongation of her dissociative identity disorder maintained her original personality at a dormant state. In fact, the memory rupture and amnesia are well recognized in dissociative identity disorder and did not occur with personalities (B) and (C), which retained almost all the memories of the other personalities.

## 3. Discussion

This is the first report of EDS leading to transvestism, which is rarely seen in clinical practice but sometimes encountered by public health nurses. This study may contribute toward expanding the perspective of medical practitioners. The findings of these rare cases may be limited in terms of generalizability but is worthy of publication from a scientific perspective. As EDS involves uncomfortable or even distressing symptoms, the two patients could have experienced incongruent feelings in personality as well as gender identity.

In this case report, the causative genes were not identified in each patient through genetic testing; instead the diagnoses were performed based on typical clinical symptoms. The difference in intellectual level between the two patients may have led to different phenotypes, hypochondriac behavior in Case 1 and dissociative identity disorder in Case 2. Both Case 1 and Case 2 presented with complications involving mood disorders and transvestism, although their clinical symptoms were both unique and different. The most notable characteristic of these patients was transvestism, while they received entirely different diagnoses based on the DSM-5. Patients with EDS tend to develop mood disorders due to pain and other reasons, such as bullying ensuing from their physical limitations [24] and psychological distress imposed by their parents as observed here. While patients with EDS reportedly present mood disorder-associated complications, the precise pathology and diagnosis of mood disorders, frequently occurring in EDS, have not been fully elucidated. The patients with EDS reported here had bipolar disorder and dysthymia, respectively, and the manic symptoms observed in Case 1 could have been inherited from the father. Physical symptoms, such as pain and injuries by EDS, bullying, and excessive interference from parental control, were present in both cases and are commonly associated with mood disorders. Although the patient in Case 1 had been molested by his father, no report was found for a direct association between sexual trauma and transvestism. In a similar case report by another study, a male employee with intellectual developmental disorder was repeatedly inappropriately touched against his wishes by his boss, although there was no sexual contact. Thereafter he started cross-dressing and constantly feared that men in general might try to “rape” or assault him [19]. In that case, the transvestic behavior was associated with anxiety and obsessive thoughts that are not necessarily related to sexual events and could be more noticeably related to intellectual developmental disorder.

In Case 2, the patient's monozygotic twin sister also had EDS, but not dissociative developmental identity, transvestism, or gender dysphoria. The fact that not both monozygotic twin sisters exhibited cross-dressing behavior could be explained by their diverse psychological experiences, not by biological mechanisms. We, however, have no detailed psychological information about her twin sister, except the patient's role, acknowledged by herself and others, as sort of elder sister who takes care of her sister in her family. The patient reported feelings of distress and irritation pertaining to her parents' attempts to control her, especially regarding decision making at home and in social activities. Her cross-dressing behavior is more likely to indicate gender incongruity and less likely transvestic behavior, because her feelings of gender identity mismatch caused significant anxiety, depression, and despair. Thus, there was a mixed effect of dissociative identity disorder and gender dysphoria on her cross-dressing. Considering the relationship between dissociative identity disorder and cross-dressing behavior in Case 2, the feeling of mismatched sex may have strongly influenced her gender identity and might have led to the development of two or more distinct personality states employed to escape from the identity issue. Taken together, since the patient in Case 2 had comorbid dissociative identity disorder and gender dysphoria, she was somewhat satisfied to experience being a man through the male personalities without desiring an actual sex-change operation.

## 4. Conclusion

Psychological issues and mood disorders often complicate the course of patients with EDS. Medical professionals should be aware that these patients are likely to have diverse secondary psychiatric symptoms and hypoactivity associated with physical, social, and psychological distress. The association with the negative aspects of EDS affects the patients' social lives and dimensional identification issues. Based on the multidimensional nature of quality of life, medical professionals need to pay special attention to patients with EDS regarding evaluation and treatment. It is necessary to consider the patient's social and psychological stress based on individual background for the purpose of EDS genetic counseling, as well as for medical management of psychiatric symptoms of EDS.

## Abbreviations

EDS: Ehlers-Danlos Syndrome
DSM-5: Diagnostic and Statistical Manual of Mental Disorders, 5th Edition
IQ: Intelligence Quotient
CSP: Cavum Septi Pellucidi.
